# A Delphi Study Protocol to Identify Recommendations on Physical Activity and Exercise in Patients with Diabetes and Risk of Foot Ulcerations

**DOI:** 10.3390/ijerph182010988

**Published:** 2021-10-19

**Authors:** Alba Gracia-Sánchez, Adriana López-Pineda, Esther Chicharro-Luna, Vicente F. Gil-Guillén

**Affiliations:** 1 Department of Behavioral and Health Sciences, Miguel Hernández University, 03550 San Juan de Alicante, Spain; agracia@umh.es (A.G.-S.); ec.luna@umh.es (E.C.-L.); 2 Clinical Medicine Department, Miguel Hernandez University, 03550 San Juan de Alicante, Spain; vte.gil@gmail.com

**Keywords:** diabetic foot, diabetic neuropathies, exercise, physical activity

## Abstract

Patients with diabetes mellitus are exposed to important complications, such as diabetic neuropathy or peripheral vascular disease. The evidence on the guidelines that these patients, with a certain risk of suffering foot ulcerations, should follow before, during and after physical exercise is scarce. The objective of this study is to identify the physical exercise guidelines to recommend based on the risk of the foot of the patient with diabetes through a consensus of experts. A three-round Delphi study will be conducted. A scientific committee (multidisciplinary group of four national experts) will review the proposal of experts and the Delphi questionnaire before submitting. A group of experts in the management and approach of the diabetic foot of an international and multidisciplinary nature will form the panel of experts, who must express their degree of (dis)agreement with each of the statements contained in the Delphi questionnaire. The percentage will be calculated in response categories, and a cut-off point of 80% will be set to define the consensus of (dis)agreement of the panelists. The results of the study could provide a series of recommendations on the realization of physical exercise in diabetic patients at risk of suffering foot ulcerations.

## 1. Introduction

Diabetes mellitus (DM), one of the most common metabolic disorders, had a global prevalence of 8.4% in adults aged 18–99 years in 2017, and it is predicted to rise to 9.9% in 2045 [1]. In Spain, the prevalence of type 2 DM adjusted for age and gender was 13.8% among those aged over 18 years [2], thus corresponding to more than 5.3 million. Type 1 DM accounts for 1–5% of all people with diabetes [3]. According to the latest edition of the International Diabetes Federation Atlas [1], if this trend continues, by 2045, 628.6 million people aged 20–79 years worldwide will have DM. DM patients suffer from major complications, such as diabetic neuropathy or peripheral vascular disease [4], that can lead to diabetic foot syndrome [5].

Diabetic foot ulcers are among the most serious, costly, and alarming complications that compromise the survival and quality of life of patients with DM [4]. An estimated 15–25% of patients with diabetes develop at least one foot ulcer in their lifetime [6]. The etiopathogenesis of these foot lesions is multifactorial and can be related to neuropathy (neuropathic ulcer), peripheral vascular disease (ischemic ulcer), or both (neuroischemic ulcer), although the ultimate etiopathogenic pathway can involve a combination of these primary risk factors and other causal factors [7]. However, about 85% of diabetic ulcers are associated with neuropathy [8]. Diabetic neuropathy is a unique neurodegenerative disorder of the peripheral nervous system that preferentially targets sensory axons, autonomic axons and later, to a lesser extent, motor axons. How diabetes mellitus targets sensory neurons remains debated [9]. An increase in the pressure applied on the foot combined with loss of protective sensation leads to tissue damage and skin lesions, mainly traumatic, breaking the skin barrier and providing an entry point for microorganisms. These microorganisms can infect the ulcer and reach the bone tissue, delaying the ulcer healing process and sometimes even leading to foot amputation [10]. An estimated 50–70% of all lower limb amputations are due to diabetes. Therefore, DM is the main cause of non-traumatic lower limb amputation in Western countries, with diabetic patients being at a 15-fold higher risk than patients without diabetes [11].

Some studies reported physical activity and exercise as effective interventions to reduce the risk of diabetic foot [12]. It is important that these patients engage in physical exercise; however, exercising without control or supervision can be dangerous for these patients. The current American Diabetes Association recommendations for physical activity [13] include exercise types, intensity, duration, frequency, and progression. However, these do not include specific advice for patients with neuropathy. The recommendations suggest that those with type 2 DM should complete three or more exercise sessions per week at moderate to vigorous intensity for a total exercise time per week of more than 150 min [13]. These patients should avoid two or more consecutive days without exercise. However, the optimal intensity of physical activity for DM patients remains unclear and controversial [13].

On the other hand, the International Working Group on the Diabetic Foot (IWGDF) [10] identified five key elements to preventing foot ulcers: identification of the foot at risk; regular inspection and examination of the foot at risk; education of patients, their family, and healthcare professionals; and managing the risk factors for ulceration [10]. These guidelines concluded that the risk stratification for diabetic foot should be performed annually in all DM patients to identify signs or symptoms of loss of protective sensation and peripheral arterial disease to determine whether the risk of developing a foot ulcer has increased [10]. It is also recommended that physicians screen DM patients who are at any risk of developing a foot ulcer (IWGDF risk 1–3), particularly those with a history of foot ulcers or lower-extremity amputation, a diagnosis of end-stage renal disease, the presence or progression of foot deformity, limited joint mobility, the presence of abundant calluses, and any pre-ulcer signs on the foot [10].

Although the guidelines recommend exercise for DM patients as a tool to prevent and control the disease course, there is scarce information about exercise for individuals with diabetic peripheral neuropathy in addition to other chronic complications associated with sustained hyperglycemia, and its effect on patients’ feet and the development of foot ulcers is unclear. Therefore, doubts arise about exercise type and intensity as well as safety for patients at high risk of ulceration or re-ulceration. We hypothesize that the recommendations agreed upon by a multidisciplinary and international experts could help professionals to recommend exercise in these patients, taking into account the different risk factors they present. As clear agreement is lacking among researchers regarding recommendations for these patients before, during, and after exercise, it is important to reach consensus among experts on the subject. Therefore, the objective of the present study will be to reach a consensus on the recommendations of exercise, according to the risk of foot ulcers in people with diabetes.

## 2. Materials and Methods

### 2.1. Study Design

An observational, cross-sectional and descriptive study based on a Delphi method [14] will be carried out to pursue the study objective. This method was used over recent years among experts in several areas of knowledge, with the results showing a high degree of agreement [15,16,17]. It is an appropriate methodology in cases involving a lack of agreement, incomplete knowledge, uncertainty, or lack of evidence [18]. It is a systematic and interactive process for obtaining the opinions and, if possible, the consensus of a group of experts. This method maintains participant anonymity from start to finish, thus allowing controlled feedback [19]. This study will be performed according to the guidelines established by CREDES (conducting and reporting of Delphi studies) [20].

### 2.2. Participants

This study will include two types of participants.

#### 2.2.1. Scientific Committee

The scientific committee will consist of a multidisciplinary group of four national experts in the field of diabetic foot, including two physicians specializing in endocrinology, a podiatrist, and a physiotherapist. All must have completed doctoral studies and have demonstrable clinical experience in treating diabetic foot. Publications in diabetic foot management and membership in an association or research group involved in diabetic foot prevention will also be valued. The physiotherapist must meet the same criteria mentioned above, except for clinical experience in treating diabetic foot since this healthcare professional does not have such clinical skills and is not included in the group of professionals forming the diabetic foot unit. Therefore, knowledge in this area through publications, presentations at conferences or training courses given in the field of diabetic foot will be valued.

The responsibilities of the scientific committee should be fully defined from the start of the study, and the members of the committee should be informed of their functions. This committee is responsible for reviewing the first version of the questionnaire created by the research team and for suggesting modifications until the version is sent to the expert panel. It is also responsible for reviewing the criteria used to select experts participating in the Delphi consensus and suggesting new members, provided that they meet the selection criteria.

An invitation letter shall be sent to the professionals proposed by the research team to form part of the scientific committee. Once they agree to participate in the study, each member of the committee shall individually receive the documents to be reviewed by email. Initially, they shall have 20 days to review the documents, a period of time that can be extended if necessary.

#### 2.2.2. Expert Panel (Panelists)

The expert panel shall provide their opinion on the items included in the Delphi questionnaire in an individual and anonymous manner. The expert panel shall be international and multidisciplinary. Managing diabetic foot requires a multidisciplinary approach; thus, selecting only one medical specialty would not cover all of the study objectives.

The expert panel can consist of professionals from different disciplines (endocrinologists, family physicians specializing in diabetes, vascular surgeons, podiatrists, physiotherapists, and physical activity and sports professionals). The experts can be national or international, and the selection criteria will differ according to their discipline:

1. Selection criteria for podiatrists and podologists:

All participants must have doctoral studies, specialization studies in diabetic foot, and scientific publications in that field. Providing training in the field of diabetic foot, giving lectures or participating in conferences, membership in an association or research group in the field of diabetes, and working or having worked in units specializing in diabetic foot in Spain or abroad will be valued.

2. Selection criteria for endocrinologists, family physicians, and vascular surgeons:

All participants should have specialized studies and clinical experience in diabetic foot as well as scientific publications in that field. Doctoral studies, providing training in the field of diabetic foot, giving lectures or participating in conferences, membership in an association or research group in the field of diabetes, and working or having worked in units specializing in diabetic foot in Spain or abroad will be valued.

3. Selection criteria for experts in physical activity and sports sciences and physiotherapists:

All participants should have completed doctoral studies and be university lecturers. Membership in an association or research group in the field of diabetes as well as recent research in sports and diabetes or sports management in patients with chronic diseases will be valued. Recent publications in the field under study will be valued.

The potential panelists shall be identified by the research team through a search of different research groups in the field of study and among the authors of recent publications on the subject. The scientific committee shall review that list and may suggest additional experts. Those who meet the eligibility criteria will be invited by email to participate in the study as a member of the expert panel without a requirement for their physical presence.

### 2.3. Delphi Method

#### 2.3.1. Data Collection Instrument

The opinion of the expert panel on the subject of this study shall be collected using a Delphi questionnaire consisting of statements (negative or positive) grouped into thematic blocks on which the participants indicate their degree of agreement or disagreement. The questionnaire’s content is based on a previous literature review and the experience of the research team and the scientific committee. The research team shall send the first version of the questionnaire to the members of the scientific committee, who will review it individually, make suggestions, and propose modifications. Based on the contributions of the committee, the research team shall modify the questionnaire until it receives final approval.

1. Literature Review:

First, the research team shall review the current literature on the subject by searching the PubMed, Scopus, and Cochrane Library databases. The most recently published meta-analyses, systematic reviews, and randomized clinical trials shall be selected.

Based on the results of the literature review, the research team shall set the items for the first version of the Delphi questionnaire to be subsequently reviewed by the scientific committee. The Delphi questionnaire will be the same for all participants regardless of their specialty.

2. Questionnaire Format:

The questions used in the Delphi process cannot be open ended since this would prevent their subsequent statistical analysis. The questions will have an appropriate format for a numerical and objective integration of the collected responses [20]. Accordingly, three main thematic blocks shall be created: recommendations prior to, during, and after physical activity. The four degrees of risk for diabetic foot shall be addressed within each block through a series of items. An item may consist of a single statement (positive or negative), or of several statements associated with the main item.

**Figure 1:**
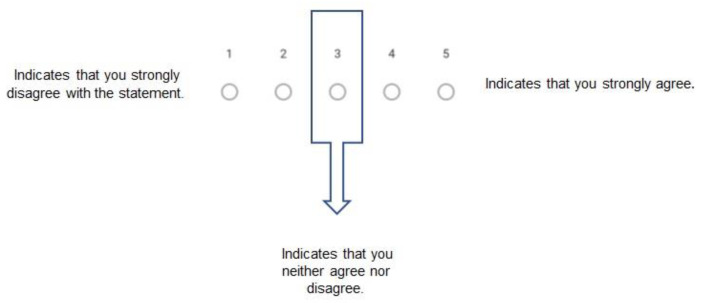
Visual scale Delphi questionnaire.

The response consists of a visual scale in which experts can indicate their degree of agreement or disagreement with the statement, according to the following defined scale: 1 represents strong disagreement with the statement, while 5 represents strong agreement with the statement. If they are unsure about their response, they will be asked to indicate number 3, thus stating that they neither agree nor disagree with the statement (Figure 1).

If the expert does not agree with any of the statements proposed for a main item, a free text space shall be added for contributions or clarifications regarding any aspect of the item that was not covered, if appropriate. This will allow the experts to reflect on their opinion in writing; this option will only be offered in the first round of the questionnaire.

Google forms shall be used to prepare the online questionnaire. The expert panel can modify their responses at any time until submission. The responses are automatically saved in a spreadsheet at the end of each round. Prior to the data analysis, a member of the research team will download the spreadsheet and delete the respondents’ identifying data. The responses shall be coded by panelist specialty and a consecutive number, e.g., podiatrist 1, endocrinologist 2, physical therapist 3.

#### 2.3.2. Delphi Process

The Delphi technique has four main characteristics: anonymity among participants, iteration with controlled feedback of the group’s opinion, statistical aggregation of the group’s response, and expert input [21]. Anonymity allows opinions to be expressed and changed privately [22]. The iteration with controlled feedback allows ‘communication’ between participants and the sharing of perspectives [18] in addition to allowing participants to change their minds [18].

This Delphi study shall be conducted in three rounds. The selected experts will be contacted via email. They shall then receive an introductory letter according to the guidelines proposed by Jon Landeta, and the following points will appear in the letter [20]:

- Study objectives.
- Methodology.
- Candidate types and criteria for their selection.
- Number of questionnaires to be completed and approximate time for each.
- Approximate process duration.
- Potential use of the collected information.
- Benefits of and objectives for participation (final report).

An electronic link to the questionnaire will be included in the introductory letter to expedite the process. Before responding to the items in the Delphi questionnaire, the experts will first respond to a question to accept or decline their participation. Those who accept can complete the rest of the questionnaire.

1. First Questionnaire Round:

This first questionnaire round is the longest of the three since those items reaching expert consensus will be removed from the second-round questionnaire. To ensure that the Delphi questionnaire and process are adequate and do not create any problems during the study, this first round will be divided into two parts, the first being a pilot study. A total of 10 experts will be selected at random for the pilot study in the first Delphi round. They will receive an invitation to participate in the study together with the questionnaire and will be given 10 days to respond. Up to three reminders will be sent within the deadline set for the first round for having more participants. Subsequently, the research team shall assess whether the pilot study process is adequate and whether any of the participants have any problems with the questionnaire. After the process is verified as correct, the invitation and questionnaire for the first round will be sent to the rest of the selected experts. The procedure will be the same as in the pilot study if it was successful or will vary depending on the problems detected. An 8-week period is estimated to be necessary for the first round.

The data will be analyzed after collection. The items that do not reach expert consensus will be advanced to the second round. Items that do not reach sufficient consensus can be rewritten without changing their meaning considering the explanatory comments that the experts may add in the free text fields.

2. Second Questionnaire Round:

The experts who participated in the first round will be invited to respond to the Delphi questionnaire again. It will be important to remind the experts that the new version of the questionnaire will be similar to the one that they completed in the first round after the elimination of items that already reached consensus. Items can be clarified as necessary.

A report of the results of the first round will be sent together with the second round of the questionnaire to provide feedback to the experts with the mean values of the responses of the entire expert panel. The information provided to experts between one questionnaire and the following shall be an overview, with total anonymity preserved among the experts from start to finish (e.g., 40% stated that…). This anonymity has the purpose of promoting response honesty since the experts can respond without being conditioned by the response of another member. It also gives them the freedom to retract their initial response at any time if considered appropriate without the other experts being aware of it. They will be given 15 days to respond to the second round of the questionnaire, and up to three reminders shall be sent within this period to obtain the highest possible response rate.

A period of 2 weeks is estimated to be necessary for the second round. The collected responses will be then analyzed, and the third-round questionnaire will be generated keeping only those items for which consensus has not been reached.

3. Third Questionnaire Round:

This is the last round of the Delphi process. The experts who responded to the previous two rounds will receive the new version of the questionnaire together with the results of the second round for soliciting feedback. The deadline for responses shall be the same as in the previous round. The results will be analyzed again once the responses are collected.

**Figure 2:**
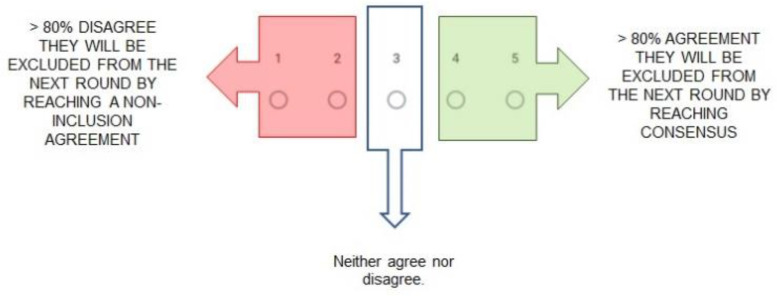
Diagram of the Delphi process.

The research team shall prepare a final report of the consensus reached at the end of the three rounds, which will be sent to the members of the scientific committee and the expert panel to thank them for their participation and collaboration in the study. It will be sent only to the experts who participate from start to finish and exclude all experts who drop out in any round of the study or decide not to participate. Figure 2 shows a diagram of the Delphi process.

#### 2.3.3. Defining and Reaching Consensus

When processing typical Delphi responses, it is necessary to differentiate them according to question type, and, therefore, response type. The partial and final results are likely to show how the level of convergence exhibited by the expert panel in each iteration varies with each question; thus, the question and not the expert panel will determine the optimal number of rounds for each individual problem [20]. The Delphi questionnaire of this study shall be composed of rating-type statements, so the percentage in response categories will be calculated in this case and a cut-off point will be set to define the (dis)agreement consensus of the expert panel. The items that do not reach consensus will advance to the following round.

### 2.4. Sample Size and Statistical Methods

#### 2.4.1. Sample Size

A minimum sample size was not calculated, as this study will use qualitative research techniques. The participants of a Delphi study should have experience and knowledge of the problem under analysis. Agreement is currently lacking on the number of experts, and there are no criteria to judge a chosen sample size [23]. According to sample size recommendations for a homogeneous sample [18], a sample size of 20–24 panelists per group is estimated to meet the study objectives, and many include fewer than 20 people [24,25,26]. However, in heterogeneous samples, as is the case in the present study, agreement is lacking on the optimal size of a Delphi panel.

The advantage of a larger panel is a more representative coverage of experts, but a corresponding disadvantage is that a panel with more than 40 or 50 participants impairs the group discussion inherent to the process [27]. Recent Delphi studies with multidisciplinary and international panels recruited fewer than 60 panelists [15,27]; others had panels with fewer than 40 panelists [28]; and multidisciplinary panels recruited as few as 19 experts [16]. Considering the sample of recent multidisciplinary and international consensus studies, our selection shall initially include a minimum of 40 experts, estimating losses or dropouts over the course of the experiment.

#### 2.4.2. Statistical Analysis

First, a descriptive analysis shall be performed by calculating frequencies and percentages of the specialty and country of each member of the expert panel.

**Figure 3:**
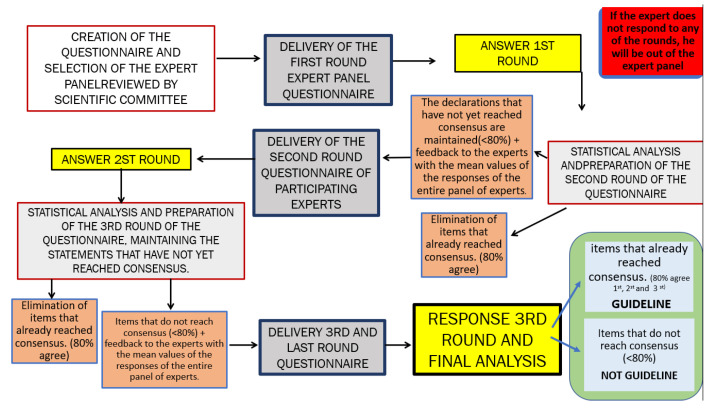
Delphi agreement diagram.

In the Delphi method, the results are analyzed independently in each round. The percentages of responses shall be calculated in categories following the consensus analysis method of most studies that also adopt the visual scale of 1–5, according to CREDES [20]. The percentages of agreement and disagreement shall be calculated for each of the items in the questionnaire, considering values of 4 and 5 as agreement and 1 and 2 as disagreement. When the percentage is greater than or equal to 80%, the statement is considered to have reached (dis)agreement consensus. The items that reach consensus are excluded from the next round (Figure 3). Finally, those items that have not reached 80% agreement or disagreement will be included to the questionnaires of the following rounds with the modifications proposed by the panelists, if they consider it necessary.

## 3. Discussion

Through an expert consensus, physical exercise guidelines based on the risk of the foot of the diabetic patient will be developed. This will be the first set of physical activity recommendations for DM patients, according to their risk of foot lesions and agreed upon by a multidisciplinary international panel. These recommendations will address different issues of the foot and patient condition before, during and after exercise. Recent publications discussed the beneficial effects of physical exercise in diabetic patients with risk factors for ulceration [12,29]. However, there are no clear exercise guidelines or recommendations for DM patients based on diabetic foot risk. We believe that establishing clear and consensual recommendations could have many advantages for these patients, including adherence to exercise since many are afraid to exercise for fear of ulceration.

The results of this study should be interpreted considering the limitations of the qualitative studies. To ensure the correct interpretation of the items of the questionnaire by the participating experts, the first version shall be reviewed by the scientific committee, which can suggest improvements, and a pilot study shall be performed in the first round with 10 of the selected experts. Another possible limitation is the use of a questionnaire with closed-ended responses. However, a free text space will be included so the experts can express their points of view. Another inherent limitation of this type of study is the experts’ voluntary participation, which could create selection bias.

## 4. Conclusions

This study is expected to provide a set of guidelines and recommendations for physical activity and exercise in type 1 and type 2 DM patients based on their diabetic foot risk. Similarly, this study can be a starting point for the development of different studies with more scientific evidence that can later help establish the safety and efficacy of different physical exercise interventions in these patients. As this is an international and multidisciplinary consensus, in clinical practice, this guide might be useful for professionals of different specialties in continuous contact with diabetic patients, improving the safety of the patient and the scope.

## References

[1] Cho N.H., Shaw J.E., Karuranga S., Huang Y., da Rocha Fernandes J.D., Ohlrogge A.W., Malanda B. (2018). IDF Diabetes Atlas: Global estimates of diabetes prevalence for 2017 and projections for 2045. Diabetes Res. Clin. Pract..

[2] Soriguer F., Goday A., Bosch-Comas A., Bordiú E., Calle-Pascual A., Carmena R., Casamitjana R., Castaño L., Castell C., Catalá M. (2012). Prevalence of diabetes mellitus and impaired glucose regulation in Spain: The Di@bet.es Study. Diabetologia.

[3] Rojo-Martínez G., Valdés S., Soriguer F., Vendrell J., Urrutia I., Pérez V., Ortega E., Ocón P., Montanya E., Menéndez E. (2020). Incidence of diabetes mellitus in Spain as results of the nation-wide cohort di@bet.es study. Sci. Rep..

[4] Jeon B.J., Choi H.J., Kang J.S., Tak M.S., Park E.S. (2017). Comparison of five systems of classification of diabetic foot ulcers and predictive factors for amputation. Int. Wound J..

[5] O’Loughlin A., McIntosh C., Dinneen S.F., O’Brien T. (2010). Review paper: Basic concepts to novel therapies: A review of the diabetic foot. Int. J. Low. Extrem. Wounds.

[6] Zhang P., Lu J., Jing Y., Tang S., Zhu D., Bi Y. (2017). Global epidemiology of diabetic foot ulceration: A systematic review and meta-analysis†. Ann. Med..

[7] Pérez-Panero A.J., Ruiz-Muñoz M., Cuesta-Vargas A.I., Gónzalez-Sánchez M. (2019). Prevention, assessment, diagnosis and management of diabetic foot based on clinical practice guidelines: A systematic review. Medicine.

[8] Wang F., Zhang J., Yu J., Liu S., Zhang R., Ma X., Yang Y., Wang P. (2017). Diagnostic Accuracy of Monofilament Tests for Detecting Diabetic Peripheral Neuropathy: A Systematic Review and Meta-Analysis. J. Diabetes Res..

[9] Feldman E.L., Callaghan B.C., Pop-Busui R., Zochodne D.W., Wright D.E., Bennett D.L., Bril V., Russell J.W., Viswanathan V. (2019). Diabetic neuropathy. Nat. Rev. Dis. Prim..

[10] Schaper N.C., van Netten J.J., Apelqvist J., Bus S.A., Hinchliffe R.J., Lipsky B.A. (2020). Practical Guidelines on the prevention and management of diabetic foot disease (IWGDF 2019 update). Diabetes. Metab. Res. Rev..

[11] Leone S., Pascale R., Vitale M., Esposito S. (2012). Epidemiology of diabetic foot. Le Infez. Med. Riv. Period. Eziologia Epidemiol. Diagn. Clin. Ter. Delle Patol. Infett..

[12] Matos M., Mendes R., Silva A.B., Sousa N. (2018). Physical activity and exercise on diabetic foot related outcomes: A systematic review. Diabetes Res. Clin. Pract..

[13] Colberg S.R., Sigal R.J., Yardley J.E., Riddell M.C., Dunstan D.W., Dempsey P.C., Horton E.S., Castorino K., Tate D.F. (2016). Physical activity/exercise and diabetes: A position statement of the American Diabetes Association. Diabetes Care.

[14] Humphrey-Murto S., Varpio L., Wood T.J., Gonsalves C., Ufholz L.A., Mascioli K., Wang C., Foth T. (2017). The Use of the Delphi and Other Consensus Group Methods in Medical Education Research: A Review. Acad. Med..

[15] Sudore R.L., Lum H.D., You J.J., Hanson L.C., Meier D.E., Pantilat S.Z., Matlock D.D., Rietjens J.A., Korfage I.J., Ritchie C.S. (2017). Defining Advance Care Planning for Adults: A Consensus Definition From a Multidisciplinary Delphi Panel. J. Pain Symptom Manag..

[16] Dribin T.E., Sampson H.A., Camargo C.A., Brousseau D.C., Spergel J.M., Neuman M.I., Shaker M., Campbell R.L., Michelson K.A., Rudders S.A. (2020). Persistent, refractory, and biphasic anaphylaxis: A multidisciplinary Delphi study. J. Allergy Clin. Immunol..

[17] Arriero-Marín J.M., Orozco-Beltrán D., Carratalá-Munuera C., López-Pineda A., Gil-Guillen V.F., Soler-Cataluña J.J., Chiner-Vives E., Nouni García R., Quesada J.A. (2021). A modified Delphi consensus study to identify improvement proposals for COPD management amongst clinicians and administrators in Spain. Int. J. Clin. Pract..

[18] Trevelyan E.G., Robinson N. (2015). Delphi methodology in health research: How to do it?. Eur. J. Integr. Med..

[19] El metodo delphi: Landeta, Jon: Libros. https://www.amazon.es/El-metodo-delphi-Jon-Landeta/dp/8434428369.

[20] Jünger S., Payne S.A., Brine J., Radbruch L., Brearley S.G. (2017). Guidance on Conducting and REporting DElphi Studies (CREDES) in palliative care: Recommendations based on a methodological systematic review. Palliat. Med..

[21] Keeney S., Hasson F., McKenna H.P. (2001). A critical review of the Delphi technique as a research methodology for nursing. Int. J. Nurs. Stud..

[22] Toronto C. (2017). Considerations when conducting e-Delphi research: A case study. Nurse Res..

[23] Jandhyala R. (2020). Delphi, non-RAND modified Delphi, RAND/UCLA appropriateness method and a novel group awareness and consensus methodology for consensus measurement: A systematic literature review. Curr. Med. Res. Opin..

[24] Akins R.B., Tolson H., Cole B.R. (2005). Stability of response characteristics of a Delphi panel: Application of bootstrap data expansion. BMC Med Res. Methodol..

[25] de Villiers M.R., de Villiers P.J.T., Kent A.P. (2005). The Delphi technique in health sciences education research. Med. Teach..

[26] Marti-Martinez L.M., Gracia-Sánchez A., Ferrer-Torregrosa J., Lorca-Gutierrez R., Garcia-Campos J., Sánchez-Pérez S.P. (2019). Description of the surgical technique for condylectomy with minimally invasive surgery to treat interdigital helomas on the lesser toes: A Delphi study. J. Foot Ankle Res..

[27] Bishop D.V.M., Snowling M.J., Thompson P.A., Greenhalgh T., Adams C., Archibald L., Baird G., Bauer A., Bellair J., Boyle C. (2016). CATALISE: A multinational and multidisciplinary Delphi consensus study. Identifying language impairments in children. PLoS ONE.

[28] Okoli C., Pawlowski S.D. (2004). The Delphi method as a research tool: An example, design considerations and applications. Inf. Manag..

[29] Liao F., An R., Pu F., Burns S., Shen S., Jan Y.-K. (2019). Effect of Exercise on Risk Factors of Diabetic Foot Ulcers: A Systematic Review and Meta-Analysis. Am. J. Phys. Med. Rehabil..

